# An unusual cause of acute abdominal pain – A case presentation

**DOI:** 10.1186/1471-2326-6-1

**Published:** 2006-04-07

**Authors:** Rao V Wunnava, Trevor M Hunt

**Affiliations:** 1 Department of Colorectal Surgery, Royal Shrewsbury Hospital, Mytton Oak Road, Shrewsbury, UK

## Abstract

**Background:**

In 1983, Graham Hughes described a condition of Antiphospholipid Syndrome in which there was a danger of thrombosis. The condition is readily detectable by blood tests and, once diagnosed; the risk of further thrombosis can be significantly reduced by anticoagulation treatments. Affected groups of patients can be distinguished by a specific blood test – the detection of antiphospholipid antibody (Ref-1). Patients with Hughes syndrome have hypercoaguable state with a markedly increased risk of both arterial and venous thrombosis and there is temporal persistence of antibody positivity.

**Case presentation:**

A 44-year-old woman was admitted under the acute surgical "take" with left sided abdominal pain radiating to her back. She had a history of borderline thyrotoxicosis in the early 1990s. She was on etonogestrel-releasing implants for contraception and there was no history of previous deep venous thrombosis. She was very tender, locally, over the left side of the abdomen. Investigations showed haemoglobin of 13.2 g/dl, white cell count of 19.9 10*9/L, and platelets 214 10*9/L with neutrophilia. Amylase and renal function tests were found to be normal. Liver function tests were deranged with Gamma GT 244 u/l (twice normal). An abdominal Ultrasound Scan suggested a possible splenic infarction, which was confirmed by a CT scan of her abdomen. Tests were carried out to investigate the possibility of a post thrombotic state. Coagulation risk factors for thrombosis were within the normal limits; Protein S 67 %(60–140), Protein C 103 % (72–146), Antithrombin 3 110 %(80–120) and Activated P C Resistance was 1.9(2.0–4.3). The Hams test was negative but the Anticardiolipin antibody test was positive. IgM level was 52 (normal is up to 10) and IgG was 18.8 (normal is up to 10). She also had border line APC Sensitivity 1.9 (2 to 4.3). Kaolin time 49 sec (70–120) Ktmix 64 sec (70–120), thyroid function test revealed TSH 0.32 mu/L, fT4 20.2 pmol/L (10–25). Subsequent determination of Anticardiolipin antibody was negative. Her symptoms were settled with the use of simple analgesia and she was discharged home with long-term anticoagulation medication. The INR target for long-term anticoagulation was aimed at >3.

**Conclusion:**

This case presented to us as an acute abdominal pain. Subsequent investigations revealed the presence of splenic infarction. Coagulation risk factors for thrombosis proved negative. Haematological investigations revealed the presence of anticardiolipin antibodies at the first instance but subsequent determinations were negative. Hence, it mimicked Hughes syndrome initially but the criteria for temporal persistence of anticardiolipin antibody was not fulfilled. Unusual surgical presentation of a thrombotic abnormality as abdominal pain due to splenic infarction.

## Background

In 1983, Graham Hughes described a condition of Antiphospholipid Syndrome in which there was a danger of thrombosis. The condition is readily detectable by blood tests and, once diagnosed; the risk of further thrombosis can be significantly reduced by anticoagulation treatments. Affected groups of patients can be distinguished by a specific blood test – the detection of antiphospholipid antibody (Ref-1). Here we report the case of a young woman presenting with acute, left-sided, abdominal pain and upon investigation, she was found to have splenic infarction. Haematological investigations were positive for anticardiolipin antibodies, but negative for coagulation risk factors for thrombosis. Interestingly her subsequent determinations for anticardiolipin antibody were also negative. She was treated with anticoagulation treatment. Patients with Hughes syndrome have hypercoaguable state with a markedly increased risk of both arterial and venous thrombosis and there is temporal persistence of antibody positivity.

## Case presentation

A 44-year-old woman was admitted under the acute surgical "take" with left sided abdominal pain radiating to her back. She worked as a dental hygienist and lived with her husband and two children. She smoked 15 cigarettes a day and there was no previous history of venous thrombosis. She had a history of borderline thyrotoxicosis in the early 1990s and underwent tension-free vaginal tape treatment for stress incontinence in September 2003. She was on etonogestrel-releasing implants for contraception. She was very tender, locally, over the left side of the abdomen but rebound tenderness was absent. Rectal examination was unremarkable. Investigations showed haemoglobin of 13.2 g/dl, white cell count of 19.9 10*9/L, and platelets 214 10*9/L with neutrophilia. Amylase and renal function tests were found to be normal. Liver function tests were deranged with Gamma GT 244 u/l (twice normal). An abdominal Ultrasound Scan suggested a possible splenic infarction, which was confirmed by a CT scan of her abdomen. Tests were carried out to investigate the possibility of a post thrombotic state. Coagulation risk factors for thrombosis were within the normal limits; Protein S 67 %(60–140), Protein C 103 %(72–146), Antithrombin 3 110 %(80–120) and Activated P C Resistance was 1.9(2.0–4.3). The Hams test was negative but the Anticardiolipin antibody test was positive. IgM level was 52 (normal is up to 10) and IgG was 18.8 (normal is up to 10). She also had border line APC Sensitivity 1.9 (2 to 4.3). Kaolin time 49 sec (70–120) Ktmix 64 sec (70–120), thyroid function test revealed TSH 0.32 mu/L, fT4 20.2 pmol/L (10–25). Subsequent determination of Anticardiolipin antibody was negative. Her symptoms were settled with the use of simple analgesia and she was discharged home with long-term anticoagulation medication. The INR target for long-term anticoagulation was aimed at >3.

## Conclusion

This case presented to us as an acute abdominal pain. Subsequent investigations revealed the presence of splenic infarction. Coagulation risk factors for thrombosis proved negative. Haematological investigations revealed the presence of anticardiolipin antibodies at the first instance but subsequent determinations were negative. Hence, it mimicked Hughes syndrome initially but the criteria for temporal persistence of anticardiolipin antibody was not fulfilled. Antiphospholipid Syndrome (APS) represents a hypercoaguable state with increased incidence of both arterial and venous thrombosis. The characteristic features include vascular thrombosis, pregnancy loss, presence of antibodies, neurological disease, thrombocytopenia and lividoreticularis. The antibodies are directed against phospholipids proteins, and there is a linear association between the tendency to clot and the level of phospholipids antibodies (Ref-2). Most of the patients are young women and potential risk factors are contraceptive pills, smoking, and hormone treatment. The gender ratio is 9 females to 1 male. Antiphospholipid antibodies are found in autoimmune disorders like Systemic Lupus Erythematosus (SLE), certain types of thyroid disease, rheumatoid arthritis, and vasculitis.

Patients taking medications such as Dilantin, Phenothizines or Hydralazine may develop Antiphospholipid Syndrome. Frequently antiphospholipid antibodies will disappear once the medication has been stopped. Antiphospholipid antibodies may also appear for a short time during a viral infection but disappear soon after the viral infection is completely resolved. Most often, however, antiphospholipid antibodies are found at the time a patient has developed a blood clot and none of the previously mentioned causes could be found.

Main symptoms of Antiphospholipid Syndrome are cerebrovascular symptoms; these can vary from mainly migraines and transient ischaemic attacks to full-blown strokes, memory loss, choreo-athetosis, myelopathy, transverse myelitis and epilepsy. Cardiovascular involvement; this includes the risk of fatal, and non-fatal, heart attacks in young women because of inflammatory thrombotic processes. Some patients develop accelerated atheromatous disease and there is high prevalence of echocardiographic valve problems.

Thrombotic complications; these can present with visceral infarctions. In kidneys, micro thrombi can form and additionally there can be evidence of renal vein thrombosis. Past case studies have shown a higher prevalence of renal artery stenosis (26 %) in patients with APS who have difficult-to-control hypertension, than in a hypertensive group with otherwise healthy potential renal donors (Ref-8). APS is also found to be a cause of Budd chiari syndrome and Addison's disease, due to acute infarction of adrenal glands, has also been reported. E. coli infections and surgery can precipitate wide spread thrombosis in these patients. One large study in the USA did CT scans on 215 patients with antiphospholipid syndrome and found splenic infarction in only six patients (Ref-9).

Current therapies used to prevent recurrent thrombosis are controversial. Anticoagulant treatment is a better option than anti-aggregants alone but there is a risk of bleeding with anticoagulant treatment necessitating the need for frequent monitoring of the INR (International normalized ratio) to measure the anticoagulant effect of the warfarin, which concerns both patients and physicians. Acute thrombosis is treated by heparin infusion followed by warfarinisation. Utilising low-molecular-weight heparin reduces the risk of thrombocytopenia. Most available data supports the long-term oral anticoagulation treatment. Prospective studies suggested that an INR between 2.0 – 2.8 might be an adequate prophylaxis level for venous thrombosis while retrospective studies suggest the need for a higher intensity anticoagulation treatment to prevent recurrent arterial events. (Ref-4). This case was an unusual surgical presentation of a thrombotic abnormality as abdominal pain due to splenic infarction.

## Competing interests

The author(s) declare that they have no competing interests.

## Authors' contributions

This Case report was prepared and drafted by Rao V Wunnava. Both authors read and approved the final manuscript.

## Pre-publication history

The pre-publication history for this paper can be accessed here:


